# High-Infiltration of Tumor-Associated Macrophages Predicts Unfavorable Clinical Outcome for Node-Negative Breast Cancer

**DOI:** 10.1371/journal.pone.0076147

**Published:** 2013-09-30

**Authors:** Yue Zhang, Shaoqiang Cheng, Mingyan Zhang, Lina Zhen, Da Pang, Qingyuan Zhang, Zhigao Li

**Affiliations:** The Affiliated Tumor Hospital of Harbin Medical University, Harbin, China; H.Lee Moffitt Cancer Center & Research Institute, United States of America

## Abstract

The tumor microenvironment is composed of tumor cells, fibroblasts, endothelial cells and infiltrating immune cells, which may inhibit or promote tumor growth and progression. The objectives of this retrospective study were to characterize the density of tumor-associated macrophages (TAMs) in breast cancer, and to correlate the density of TAMs with clinicopathological parameters. Paraffin-embedded specimens and clinicopathological data, including up to 5 years follow-up information, were obtained from 172 breast cancer patients. Immunohistochemical staining for CD68 (marker for macrophages) was performed and evaluated in a blinded fashion. We found that TAMs were significantly frequent in high histopathological grade breast cancer patients. Breast cancer patients with a high density of TAMs had significantly lower rates of disease-free survival and 5-year overall survival than patients with low density of TAMs. Furthermore, high-infiltration of TAMs indicated worse survival rate for patients with node-negative breast cancer. In conclusion, the number of TAMs in the tumor stroma is an independent predictor of survival time for breast cancer patients. High-infiltration of TAMs is a significant unfavorable prognostic factor for patients with invasive breast cancer and, as such, is a potentially useful prognostic marker for breast cancer.

## Introduction

The tumor microenvironment is comprised of tumor cells and heterogeneous populations of stromal cells such as fibroblasts, endothelial cells and infiltrating immune cells, as well as the products of these cells such as extracellular matrix, chemokines, cytokines, growth factors, enzymes and various metabolites [1], [2]. Tumor-stromal and stromal-stromal interactions have been implicated in the regulation of tumor cell growth, determining metastatic potential and the location of metastatic disease, and impacting the outcome of therapy [3]. The immune system of the tumor-bearing host interacts with tumors throughout their development [4], and the consequences of this interaction have substantial implications for cancer therapy. Among these immune cells, tumor-associated macrophages (TAMs) are considered the most powerful inhibitors of antitumor immunity and the greatest barrier to successful immunotherapy [5].

TAMs are a large component of the tumor microenvironment, comprising up to 50%∼ 80% of the tumor mass [6]. Generally, macrophages are routinely classified into two main polarized phenotypes: classically activated macrophages (M1) and alternatively activated macrophages (M2). M1 macrophages arising from exposure to the Th1 cytokines, in addition to lipopolysaccharide or endotoxin, are proinflammatory and are characterized by the production of nitric oxide synthase 2 (NOS2) and type 1 cytokines and chemokines, which are reported to have a high bactericidal and tumoricidal capacity. While M2 macrophages arising from exposure to Th2 cytokines such as interleukin (IL) 4 and IL-13. as well as IL-10, release anti-inflammatory molecules such as IL-4, IL-13 and transforming growth factor beta[7]. Although both M1 and M2 can infiltrate into tumor sites, naturally arised TAMs are biased towards the M2 type and show mostly pro-tumor functions, promoting tumor progression, inducing tumor-anginogenesis and dampening anti-tumor immune response [8], [9]. It has been well taken that high-infiltration of TAMs are correlated with a poor prognosis for most solid tumors [10]-[12]. In breast cancer, a high focal infiltration of TAMs directly correlates with tumor cell invasion, increased vascularization and axillary lymph node involvement [13], [14]. Patients with higher TAMs density have significantly worse relapse-free survival (RFS) and overall survival(OS) [15]. Recently, Catharina et al further demonstrated that the presence of TAMs in tumor stroma but not in tumor nest was an independent prognostic factor for reduced breast cancer specific survival [16]. Despite these studies, the expression of TAMs in node-negative breast cancer has not been well documented.

Breast cancer is by far the most common cancer diagnosed and the most common cause of death from cancer in women worldwide [17]. Among prognostic factors used in clinical practice to determine the type of treatment indicated for each patient, the presence of metastatic axillary lymph nodes has been shown to be the most valuable, followed by expression of hormonal receptors, human epidermal growth factor receptor 2 (HER2/neu) status, tumor size, histological subtype, tumor grade, lymphovascular invasion and proliferative rate [18]. Although the recurrence rate of node-negative breast cancer is much lower than node-positive ones, about 20%–30% of these patients will suffer recurrences and die of their disease within 10 years [19]. Despite the existence of several prognostic factors, the prediction of clinical outcome remains a challenge. For these reasons, research is ongoing to identify better or more refined tumor prognostic markers, resulting in more effective treatment choices.

We hypothesized that high infiltration of TAMs indicate a worse survival rate for node-negative breast cancer. To test our hypothesis, we used immunohistochemical staining to analyze TAM levels in patients with breast cancer and compared these data with the clinicopathological features of these patients.

## Materials and Methods

### Patients and Tissue Specimens

This study used archival material from the Department of Pathology at the Affiliated Tumor Hospital of Harbin Medical University, Harbin, China. Breast cancer tissue specimens were obtained from patients undergoing primary mastectomies at the institution from January 1, 2006 to June 1, 2007. Pathologists diagnostically examined the tumor breast tissues which were removed from the patients. The most important inclusion criterion for the patients was the presence of primary, unilateral and operable infiltrating ductal carcinomas. Among exclusion criteria were distant metastasis at the time of diagnosis, locally advanced disease, inflammatory carcinoma and synchronous bilateral breast cancer. The pathologist measured the tumors in millimeters at the largest diameter of the invasive carcinoma. All archival tumor blocks of each tumor were initially assessed by hematoxylin and eosin (H&E) staining to select a tumor block with an invasive carcinoma and to include the tumor border and a cross-sectional area as large as possible. 4-µm-thick sections from the paraffin blocks were mounted on ChenMate slides (ZSGB-Bio, Beijing, China). Morphology and protein expression were evaluated in consecutive sections. Histological classification of tumors was based on the WHO criteria (Table 1). All protocols were reviewed and approved by the Ethical Committee of Harbin Medical University. Written consent was obtained from all participating patients.

**Table 1:** Clinical characteristics of the patients with breast carcinoma.

Variables		No. of Cases
Age	Median	49
	Range	29–73
Lymph node metastasis	Negative	56
	Positeive	116
TNM stage	I	25
	II	66
	III	76
	IV	5
Histologic grade	G1	34
	G2	107
	G3	31
Tumor size	Mean	2.8
	Median	2.8
	Range	1–7
Estrogen Receptor	Negative	75
	Positeive	97
Progesterone Receptor	Negative	68
	Positeive	104

### Follow-up

Clinical and pathological records of all patients on the study were reviewed periodically. Patients were followed regularly for 5 years at the Affiliated Tumor Hospital of the Harbin Medical University. Clinical records were obtained from the follow-up department of the hospital. All patients were followed until death or the study closing date (October 1, 2012). Disease-free survival (DFS), which measured the first recurrence at any site, and overall survival (OS), which measured death from any case, were the two assessments used for prognostic analyses.

Patients were seen for history, physical examination and routine laboratory investigations once every 3 months during the first 2 years, once every 6 months for 5 years, and once a year after that. An X-ray mammography was performed once a year or when disease recurrence was suspected. A magnetic resonance imaging (MRI) scan was occasionally also performed when disease recurrence was suspected. During the follow-up period, 88 patients had disease recurrence and 55 patients died. Contralateral breast carcinoma or second malignancies were not considered to be cases of recurrent disease.

### Determination of CD68

Immunohistochemical staining was performed using the 2-step plus Poly-HRP method. Briefly, one representative section of the tissue was cut at 4 μm and placed on poly-L-lysine coated slides. The slides were deparaffinized, dehydrated, immersed in sodium citrate buffer (pH 6.0) or Tris-EDTA buffer (pH 9.0), and pretreated in a microwave oven for 10 min. This was followed by a 10-min rinse with phosphate-buffered saline (PBS). After blocking with 3% hydrogen peroxide for 10 min at room temperature, the slides were incubated at 4°C overnight with primary anti-CD68 antibody (ab125047, dilution 1∶100, Abcam, USA). Afterwards, the slides were stained with the 2-step plus Poly-HRP anti-Rabbit IgG Detection System (ZSGB-Bio, Beijing, China). After visualization of the reaction with the DAB chromogen, the slides were counterstained with haematoxylin and covered with a glycerin gel. For negative controls, the primary antibody was substituted with PBS in order to confirm the specificity of the primary antibody.

### Evaluation of immunohistochemical staining

Sections were evaluated in a blinded manner by two experienced investigators who provided a consensus opinion of stain patterns by light microscopy. CD68^+^ TAMs was estimated by counting the number of CD68^+^ TAMs in each of the 3 tissue cores from each patient tumor sample, and the mean of three counts was taken. The median value (26 CD68^+^ TAMs/tissue cores) was used to cut off the subgroups of all immunohistochemical variables in our data. The patients were then divided into two groups: TAM high-infiltration group (≥ 26 TAMs/tissue cores, n = 86) and CD68 TAMs low-infiltration group (<26 TAMs/tissue cores, n = 86).

### Statistical Analysis

All analyses were performed using the statistical software SPSS 13.0 (SPSS, Chicago, IL, USA). The correlation of tumor-associated leukocyte immunoreactivity with patients’ clinicopathological variables was analyzed by the χ^2^ test or Fisher’s exact test. The Kaplan-Meier method was used to estimate overall survival. Survival differences according to CD68 expression were analyzed by the log-rank test. The influence of variables on survival was assessed using Cox univariate and multivariate regression analysis. The risk ratio and its 95% confidence interval were recorded for each marker. P values of < 0.05 were considered statistically significant in all of the analyses.

## Results

### Clinical results

172 patients with invasive breast carcinoma were included in the study. The median age of these patients was 49 years with a range of 29–73 years. In total, 45.3% of the patients were over 50 years of age. Tumor size distribution was 32.6% for ≤ 2 cm, 61.6% for 2–5 cm, and 5.8% for > 5 cm. Among the patients, 32.6% were axillary lymph node-negative and 67.4% were axillary lymph node-positive. 25 patients (14.5%) were at stage I, 66 patients (38.4%) were at stage II, 76 patients (44.2%) were at stage III, and 5 patients (2.9%) were at stage IV. The tumors were stratified according to histological grade (WHO classification). 34 patients were classified as grade I, 107 were grade II, and the remaining patients were grade III. At the time of study completion, 54 patients (31.4%) died, and 118 patients (68.6%) were alive. Further detailed clinical information is presented in Table 1.

### Immunohistochemical pattern of TAMs in breast carcinoma

**Figure 1:**
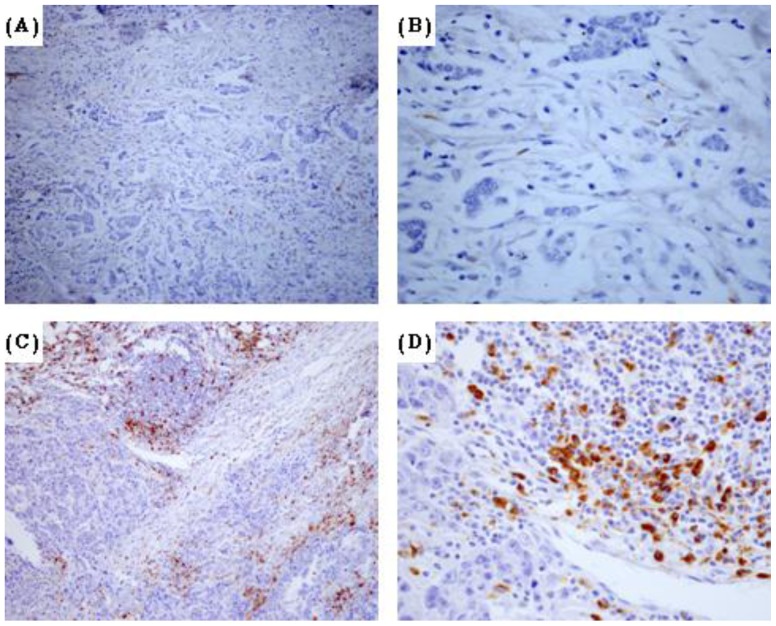
Immunohistochemical detection of tumor-associated macrophages (marker CD68) in breast cancer tissues. (A) High-infiltration specimen (x 100) (B) High-infiltration specimen (x 400) (C) Low-infiltration specimen (x 100) (D) Low-infiltration specimen (x 400).

The tumor-associated macrophages were detected using an antibody against CD68 (Figure 1), a pan-macrophage marker frequently used as a marker for TAMs [20]. The evaluation of full-face tissue sections revealed that macrophages observed in both tumor nest and stroma. Recently, several studies have showed that TAMs in tumor stroma but not in tumor nest was connected with worse survival rate for patients with breast cancer [16]. Hence, we counted the number of TAMs in three separated tumor stroma sites. The median number of CD68^+^ cells was 26 ± 13.6 cells/high-power field (HPF) and the range was 1–80 cells/HPF. To investigate the association between the density of TAMs and clinical features in breast cancer patients, the median density of CD68 positive cells was used to separate the patients into high and low groups. Of the 172 breast cancer specimens, 86 were classified as TAM high-infiltration group (Figure 1A-B), and the rest (n = 86) were TAM low-infiltration group (Figure 1C-D). TAM status was positive in 14 (41.2%) of the 34 grade I tumors, 50 (46.7%) of the 107 grade II tumors, and 22 (70.9%) of the 31 grade III tumors. These data demonstrated that the infiltration of TAMs was much higher in high-histopathological grade (G3) than in low-histopathological grade (G1 and G2) breast cancer (P = 0.024).

### Tumor-associated macrophage expressions and survival status

We used an average 5-year follow-up period to assess the survival of breast cancer patients in the context of TAM status. Patients were divided into two groups on the basis of their prognosis. The good prognosis group (n = 84) comprised patients who remained disease-free, and the poor prognosis group (n = 88) comprised patients who had recurrence, metastasis to a distant site, or had died as a result of breast cancer. As shown in Table 2, patients with a poor prognosis had a significantly higher TAM status (58/88, 59.0%) than patients who were disease-free (34/84, 40.4%) (P = 0.022).

**Table 2:** Correlation of TAM expression with prognosis.

Survival Status	No. of Cases	CD68		
		Negative	Positive	P
Good	84	50	34	
Poor	88	36	52	0.022

### Correlations between TAM status and clinicopathological features

Correlations between TAM status and various clinicopathological features are summarized in Table 3. We found no significant correlations between TAM status and age, menopausal status, tumor size, or estrogen receptor (ER) or progesterone receptor (PR) expression. CD68 infiltration, however, was significantly associated with histopathological grading (P = 0.016).

**Table 3:** Correlation between CD68 expression and various clinicopathological features.

Variables		No. of Cases	CD68		
			Negative	Positive	P
Age					
	<50	94	47	47	1.000
	>50	78	39	39	
Tumor size					
	pT1	56	34	22	0.073
	pT2-4	116	52	64	
Menopausal status					
	Premenopausal	100	48	52	0.643
	Postmenopausal	72	38	34	
Histologic grade					
	G1-2	141	77	64	0.016
	G3	31	9	22	
Lymph node status					
	N0	56	26	30	0.626
	N1-3	116	60	56	
ER status					
	Positive	97	50	47	0.759
	Negative	75	36	39	
PR status					
	Positive	104	56	48	0.275
	Negative	68	30	38	

### Kaplan–Meier survival analysis

**Figure 2:**
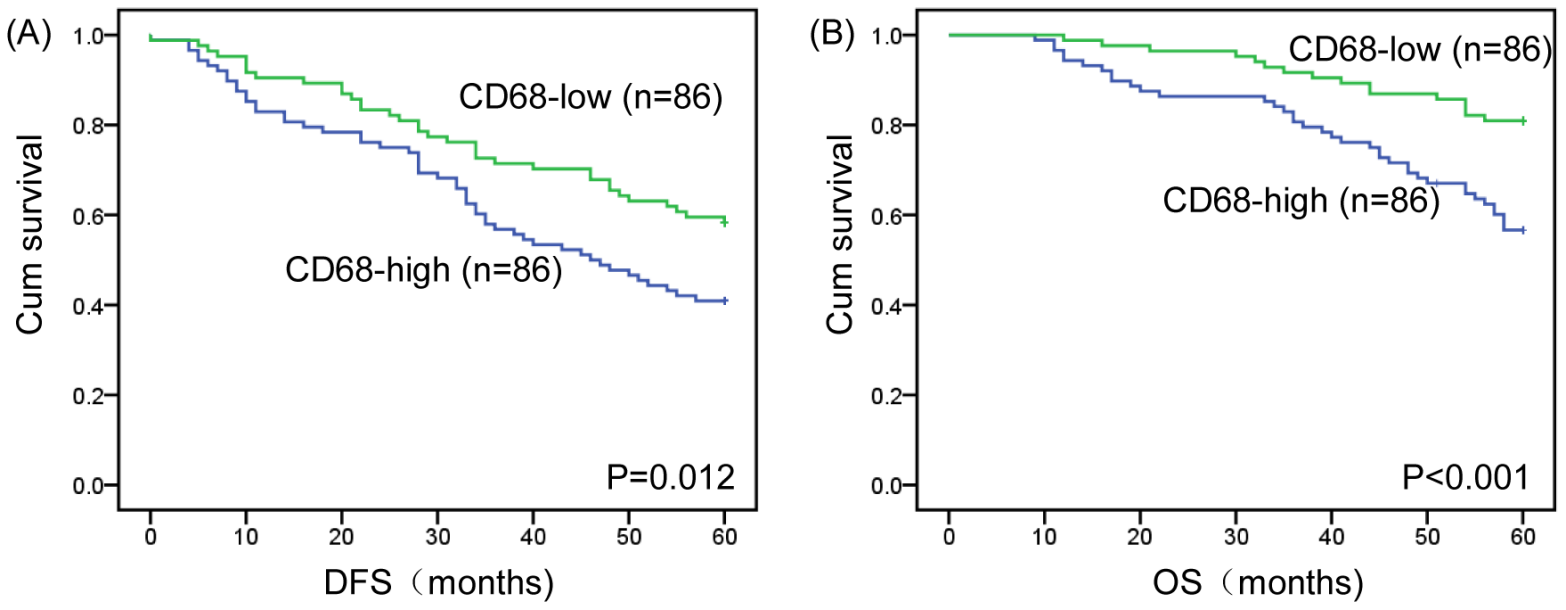
Kaplan–Meier analysis for DFS and OS based on CD68 expression in breast cancer patients. (A) Kaplan–Meier analysis for DFS based on CD68 expression in patients with breast cancer (log-rank test, P = 0.012) (B) Kaplan–Meier analysis for OS based on CD68 expression in patients with breast cancer (log-rank test, P<0.001). Green: TAM-low group; Blue: TAM-high group.

**Figure 3:**
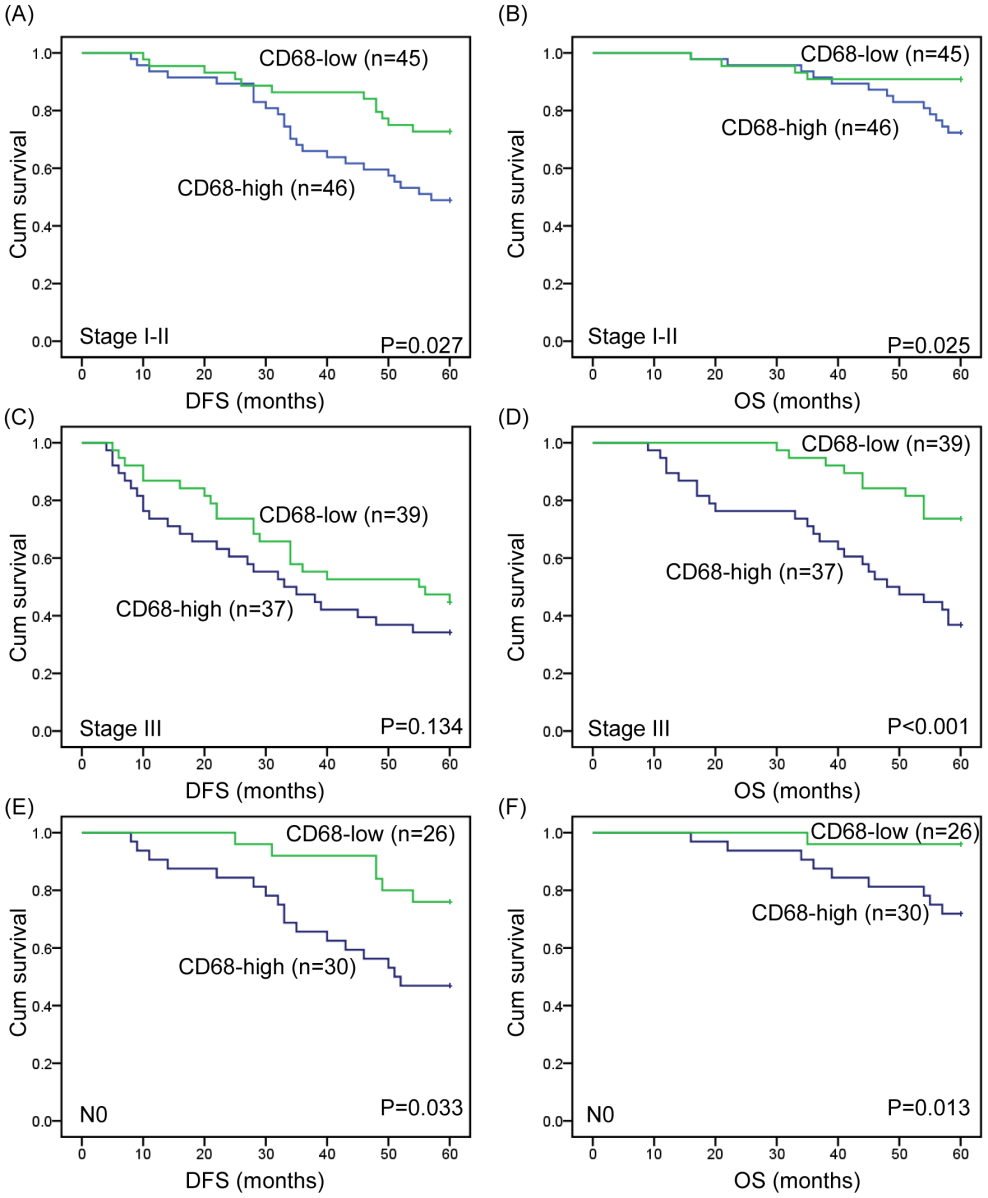
Overall survival and disease-free interval according to clinical stage and lymph node status. (A) DFS in stage I-II patients, P = 0.027 (B) OS in stage I-II patients, P = 0.025 (C) DFS in stage III patients, P = 0.134 (D) OS in stage III patients, P<0.001 (E) DFS in node-negative patients, P = 0.033 (F) OS in node-negative patients, P = 0.013. Green: TAM-low group; Blue: TAM-high group.

Kaplan–Meier survival curves are shown in Figure 2. Among the 172 study patients, TAM high-infiltration patients experienced significantly poorer outcomes in terms of overall survival (P < 0.001, log rank) and disease-free interval (P = 0.012, log rank) in comparison with patients who were TAM low-infiltration. With regard to the clinical stage, the prognosis of TAM high-infiltration cancer patients was significantly poorer than that of TAMs low-infiltration cancer patients with regard to overall survival and disease-free interval in early stage (I-II) and stage III (Figure 3A-D). More importantly, we found that for “node-negative" breast cancer patients, a high density of TAMs correlated with a significantly worse survival rate compared to patients with a low density of TAMs (figure 3E and F).

In order to eliminate the effect of adjuvant therapies on survival, we compared the treatments between the two groups. In the current study, there are 15 patients who did not receive any adjuvant therapy (including chemotherapy, radiotherapy and endocrine therapy). Among these patients, 8 patients were in TAM low-infiltration group: 3 patients are stage I; 3 patients are stage II; 2 patients are stage III. 7 patients with high TAM infiltration did not receive any adjuvant therapy: 4 patients are stage I; 2 patients are stage II; 1 patient is stage III. In addition, patients with low density of CD68 received 4.86 ± 2.59 cycles of chemotherapies, whereas patients with high density of CD68 received 5.33 ± 2.46 cycles of chemotherapies. There is no statistical significance between the two groups on aspect of adjuvant therapies (P = 0.228).

### Univariate and multivariate analyses of tumor-associated immune cell expression and clinicopathological variables

Univariate analysis of overall survival using Cox regression analysis identified high TAM infiltration (P = 0.001), histopathological grade (P = 0.010), lymph node metastasis (P = 0.009), ER status (P = 0.038) and PR status (P = 0.001) as significant prognostic predicators. Age, menopausal status and ER status had no prognostic value. Multivariate analysis was performed on the same set of patients. The results indicated that PR status (risk ratio: 0.403; P = 0.002) was an independent favorable prognostic factor. Histopathological grade (risk ratio: 2.590; P = 0.002) and lymph node metastasis (risk ratio: 0.324, P = 0.002) were independent unfavorable prognostic factors (Table 4).

**Table 4:** Prognostic factors in the Cox proportional hazards model.

Variables	Risk ratio	Univariate 95% CI	P	Risk ratio	Multivariate 95% CI	P
Age						
<50/>50	1.093	0.639–1.870	0.744	1.051	0.454–2.434	0.908
Menopausal						
Postmenopausal/Premenopausal	1.074	0.628–1.837	0.794	1.316	0.564–3.072	0.526
Histologic grade						
G1/G2-3	0.216	0.067–0.691	0.010	0.280	0.086–0.910	0.034
Tumor size						
pT1/pT2-4	0.592	0.317–1.104	0.099	1.022	0.525–1.991	0.949
Lymph node metastasis						
N0/N1-3	0.400	0.201–0.796	0.009	0.324	0.160–0.658	0.002
Estrogen Receptor						
Positive/Negative	0.568	0.333–0.970	0.038	0.668	0.383–1.164	0.154
Progesterone Receptor						
Positive/Negative	0.417	0.244–0.714	0.001	0.403	0.228–0.714	0.002
CD68						
Positive/Negative	2.643	1.473–4.742	0.001	2.590	1.429–4.695	0.002

## Discussion

In the current study, we evaluated the prognostic significance of TAMs in a large number of invasive breast carcinomas. We found that TAM migration was significantly associated with high histopathological grade. Breast cancer patients with high TAM infiltration had significantly lower DFS and 5-year survival rates than patients with low rates of TAM infiltration. In addition, for patients with negative axillary lymph nodes, a high infiltration of TAMs indicated markedly poorer survival rate than low-infiltration samples. Multivariate analysis further confirmed that an increased density of TAMs was an independent prognostic factor for patients with breast cancer.

Breast cancer is the most prevalent malignant disease in almost all countries [17]. While improvements in early detection and in adjuvant systemic therapy, have effected a decline in the mortality rate from breast cancer [21], not all populations have benefited from those advances, and breast cancer remains the leading cause of cancer death in woman in both developing and developed regions [22]. Accordingly, the development of novel drugs and strategies for the treatment of breast cancer is required, as are novel adjuvant diagnostic and prognostic biomarkers to improve treatment decisions in combination with current parameters.

The microenvironment of breast cancer is populated by many cells including fibroblasts, adipocytes and a wide range of hematopoietic cells, as well as newly formed blood and lymphatic vessels and their associated cells [23]. Among those cells, TAMs constitute a significant part of the tumor-infiltrating immune cells, and appear to play an important role in tumor progression. Macrophages are important innate immune cells with essential roles in the primary response to pathogens, normal tissue homeostasis, presentation of foreign and self-antigens following infection or injury, resolution of inflammation and wound healing [24]. Macrophages can be differentiated into either pro-inflammatory M1 macrophages or anti-inflammatory M2 macrophages. M1 macrophages are generally characterized by IL-12^high^, IL-23^high^, IL-10^low^ phenotype, which have a high bactericidal and tumoricidal capacity. By contronst, M2 macrophages generally share an IL-12^low^, IL-23^low,^ IL-10^high^ phenotype, which is involved with parasite containment, promotion of tissue remodeling and pro-tumor functions [25]. It has been known that both M1 macrophages and M2 macrophages can infiltrate into tumor microenvironment. In colorectal cancers, high density of M1 macrophages is considered to be proinflammatory and play an antitumor role, which is connected to a good prognosis [26]. Furthermore, increased activated M1 macrophages successfully blocked lung metastasis in a mammary tumor model [27]. By contrast, for most solid cancers including breast cancer, naturally accumulated TAMs seem to have a dominant M2 phenotype [28], [29]. It has been established that M2 macrophages are linked to the growth, migration and invasion of a variety of cancers [30]. In addition, they can promote tumor angiogenesis by producing angiogenic growth factors and proteinases including VEGF, matrix metalloproteinase-9 and uPA [31], [32]. More recently, new evidences have extended the repertoire of these cells to other tumor promoting activities such as interactions with cancer stem cells, response to chemotherapy and tumor relapse [33]. It seems that the balance between M1 and M2 macrophage subtypes is more valuable for predicting the survival rate for patients with breast cancer. Further investigation is needed to prove this hypothesis.

Recently, Mahmoud and colleagues [34] have confirmed the predictive value of TAMs in patients with breast cancer. In their univariate survival analysis, higher numbers of CD68^+^ macrophages predict worse overall survival and shorter disease-free interval. In this study, however, CD68^+^ macrophage count was not an independent prognostic marker in multivariate model analysis. In addition to previous studies, our data strongly indicate that high-infiltration of TAMs is an important factor in promoting tumor cell metastasis in breast carcinoma. High-infiltration of TAMs correlated with shorter survival in patients with breast carcinoma in either early or late stage, and was associated high histological grade. Furthermore, the data showed here indicated that high desity of TAM was an independent prognostic marker for patients with breast cancer. The reason for the difference between Mahmoud and our studies may be explained by choosing different sites for TAMs evaluation. Recently, several reports have demonstrated that infiltration of TAMs in tumor nest was not correlated with any clinicopathological features and did not relate to the survival rate for patients with breast cancer. By contrast, the amount of TAMs in tumor stroma was correlated with a worse prognosis [16]. Since TAMs were heterogeneous populations, we hypothesis that TAM in different of tumor sites had different phenotype and function. However, this theory need to be further investigated.

In the current study, we also evaluated the predictive value of TAMs in node-negative breast cancer patients, who form a significant percentage of breast cancer patients. In regions with widespread breast cancer screening and disease awareness among women, the rates of node-negative diseases are likely to be in the range of 65%–70% of breast cancer patients [35]. Although node–negative breast cancer patients have a better survival rate compared with node-positive patients [36], node-negative breast cancer does not automatically have a good prognosis, nor does it preclude chemotherapy. The question that remains in treatment with node-negative breast cancer today is how to properly select patients and identify the best regimen. Here, we first reported that high-infiltration TAM status was associated with a poorer survival rate for patients with node-negative breast cancer, and the TAM high-infiltration group showed a significantly worse DFS and OS than the TAM low-infiltration group. TAM infiltration therefore constitutes a new, important risk factor for node-negative breast cancer recurrence, and the question of whether node-negative patients with high TAM infiltration would benefit from more intense chemotherapy is worthy of further investigation.

In the present study, we also evaluated the correlation between TAM status and various clinicopathological features. We found that high-histopathological grade (G3) breast cancer patients had higher TAM status (23/37, 62.2%) than low-histopathological grade (G1 and G2) breast cancer patients (23/82, 28%) (Table 3) (P = 0.016). These data indicate that TAM status is significantly associated with high histopathological grade tumors, suggesting that TAM infiltration might play an important role in the process of oncogenesis.

In conclusion, high-infiltration of TAMs correlated with shorter survival in patients with breast carcinoma, and high-infiltration of TAMs was associated with high-histological grade. Our results suggest that TAMs are a negative predictor of breast carcinoma and that TAMs could emerge as an attractive new drug target in the treatment of breast carcinoma.
